# Is Consciousness First in Virtual Reality?

**DOI:** 10.3389/fpsyg.2022.787523

**Published:** 2022-02-11

**Authors:** Mel Slater, Maria V. Sanchez-Vives

**Affiliations:** ^1^Event Lab, Department of Clinical Psychology and Psychobiology, University of Barcelona, Barcelona, Spain; ^2^Institute of Neurosciences of the University of Barcelona, Barcelona, Spain; ^3^Institut d’Investigacions Biomédiques August Pi i Sunyer (IDIBAPS), Barcelona, Spain; ^4^ICREA, Barcelona, Spain

**Keywords:** consciousness, virtual reality, perception, interface theory of perception, real vs. virtual, presence

## Abstract

The prevailing scientific paradigm is that matter is primary and everything, including consciousness can be derived from the laws governing matter. Although the scientific explanation of consciousness on these lines has not been realized, in this view it is only a matter of time before consciousness will be explained through neurobiological activity in the brain, and nothing else. There is an alternative view that holds that it is fundamentally impossible to explain how subjectivity can arise solely out of material processes—“the hard problem of consciousness”—and instead consciousness should be regarded in itself as a primary force in nature. This view attempts to derive, for example, the laws of physics from models of consciousness, instead of the other way around. While as scientists we can understand and have an intuition for the first paradigm, it is very difficult to understand what “consciousness is primary” might mean since it has no intuitive scientific grounding. Here we show that worlds experienced through virtual reality (VR) are such that consciousness is a first order phenomenon. We discuss the Interface Theory of Perception which claims that in physical reality perceptions are not veridical and that we do not see the “truth” but that perception is based on evolutionary payoffs. We show that this theory may provide an accurate description of perception and consciousness within VR, and we put forward an experimental study that could throw light on this. We conclude that VR does offer an experimental frame that provides intuition with respect to the idea that “consciousness is first” and what this might mean regarding the perceived world. However, we do not draw any conclusions about the veracity of this notion with respect to physical reality or question the emergence of consciousness from brain function.

## Introduction

The standard scientific view is that consciousness will ultimately be explained as a result of brain activity. This follows the fundamental scientific paradigm that “matter” is primary and the realm of the subjective can ultimately be explained through physical laws—in this case neurobiological (Crick and Clark, 1994; Changeux, 1997; Tononi and Edelman, 1998; Rees et al., 2002; Koch et al., 2016). Hence brain activity in itself, with appeal to nothing else outside of it, can explain consciousness. Following Edelman’s classification (Edelman, 2003, 2004) this would include both primary consciousness (what we perceive) and secondary consciousness (metacognition or awareness of being aware). The “matter is primary” paradigm involves the belief that science will ultimately solve this central question for humans (Melloni et al., 2021). However, how subjectivity emerges from matter, has been a subject of intense debate by both philosophers and neuroscientists, an issue that has been referred to as the “hard problem of consciousness” (Chalmers, 1996). There is an alternative paradigm, the “primacy of consciousness” paradigm (Chalmers, 1995, 1996; Bitbol, 2008), and that, for example, the theory of physics can be derived from the study of consciousness (not the other way around) (Manousakis, 2006; Hoffman and Prakash, 2014).

The primacy of matter is so rooted in our normal way of scientific thinking that, apart from the fact that we do not yet understand how the awareness of our perceptions and feelings can arise from networks of neurons—and even though there is data relating brain activity and subjective experiences—it does not seem to be a fundamental problem: it is just a matter of time before this will be solved. However, what could the primacy of consciousness actually mean? We have no scientific experience about how the everyday world of objects, space and time and could arise from something called “consciousness.” Here we argue that virtual reality provides a paradigm for this investigation: a world that we can perceive, much as we perceive “reality,” a world that is indeed a product of consciousness.

In order to address this, first we will discuss virtual reality (VR) itself, then consider the nature of “objects” in VR, and follow this by the perception of virtual objects. We consider whether virtual objects are real in any sense, and also whether perception of virtual objects is real perception. We next discuss the Interface Theory of Perception (Hoffman et al., 2015), and how it can be useful to explain how perception in VR works, and discuss some predictions of this theory that can be tested within VR. We will pay attention to the issue of self-perception and conclude with a discussion of the implications for consciousness of ourselves and the others (self and social consciousness) and of the external world.

## Ideal-Type Virtual Reality

We could start by describing hardware and software, but it is preferable to discuss instead what VR is from the perspective of its affordances for a participant. We use the term “participant” rather than “user” since from our point of view, VR is not something you use, but a space or environment in which you are, and an environment in which you participate. We do not *use* VR any more than we are “users” of real 4D space-time. VR provides an environment, rather than a tool. It is an environment in which tools may be deployed.

An *ideal-type* immersive system in one where the participant perceives an artificial world using natural sensorimotor contingencies (O’Regan and Noë, 2001a,b). In other words, the participant looks around, listens, bends down to see underneath objects, looks around objects, turns his or her head to listen, reaches out to touch things, pushes and pulls and so on. The participant thereby can also act on objects that are perceived and can change the environment. However, what is perceived and acted upon is not the physical reality of everyday life, but an artificial reality produced by some technical means. For example, what is perceived may be computer generated using techniques of real-time computer graphics and delivered visually to the participant through stereo head-mounted display, with head-tracking that supports the participant looking and moving around, with images streamed in real-time to the displays as a function of head-tracking. Similarly, through headphones attached to the head-mounted display or through external speakers, appropriate and corresponding auditory output, even binaural sound, may be delivered. The participant may be able to act on the environment through a means of tracking his or her real body movements, thereby allowing changes to be effected, for example, through collisions between the tracked end-effectors and the locations of virtual objects. Touch and force feedback may be delivered, for example, by vibrators or by robotic devices that have information about the location of the participant’s body and movements (through the head and body tracking) and its relationship to the virtual environment, and supply just-in-time haptic feedback—for a recent implementation (see Al-Sada et al., 2020). For example, were the participant to collide with a (virtual wall) the robot would be there just at that moment to provide the needed force and tactile feedback. These are just examples.

Our description is of an ideal-type immersive system. However, every single aspect must be physically realized. For example, what is the resolution of the displays? What visual field of view do they offer? How fast and how much of the body is tracked? What actual affordances do participants have to change things? How exactly can they change things? Are their hands directly tracked or do they have to hold tracked pointing devices and only those are tracked? How do they move through the environment? Is the head tracked with 6 degrees of freedom and with the visual images updated according to head moves (translation, yaw, pitch, and roll) or only some subset of these?

It should be noted that perception through natural sensorimotor contingencies implies more than just interaction. The displays are intimately bound to this. For example, if a participant moves his or her head toward an object, the expectation will be to see the object closer and closer including its details. This might correspond to the sensorimotor contingency rule: to see an object’s details, move closer to it. But if in the act of moving closer the vision breaks down into seeing only pixels rather than detail, then this is a failure of sensorimotor contingencies. Similar arguments can be made about field-of-view, latencies, framerate, and so on. If the participant reaches out to touch something, but feels nothing in violation of expectations, then this is also a failure of sensorimotor contingencies.

Actual immersive systems can be conceptually placed in a partial order. The affordances of a VR system that supports only head rotation for tracking can be completely simulated in a VR system that supports 6 degrees of freedom head tracking—as a practical example, the affordances of an Oculus Go device can be completely simulated using an Oculus Quest, but not the other way around. We have referred to this as the 6 degrees of freedom VR being more “immersive” than the rotation only VR (Slater, 2009; Slater et al., 2010a). Similarly, a desktop-based VR system, where the participant observes the virtual environment on an external screen rather than through a head-mounted display, and controls movements through a joystick, can in principle be completely simulated by a model-based VR system experienced through a head-mounted display. The importance of this is not to say that one system is “better” than another, but only that systems at different levels of such a hierarchy can give rise to qualitatively different experiences, different qualia.

Does this mean that consciousness in a virtual world requires natural sensorimotor contingencies, i.e., that are copies of those in physical reality? In principle, this is not the case. We can consider that exploiting VR as a media means not just the possibility of reproducing our reality with high fidelity, but also allows the creation of physically impossible but virtually possible alternative worlds. Our brains do not require much time for adaptation to a virtual world perceived through natural sensorimotor contingencies—we perceive in the virtual world as usual. However, VR can be used to create new sensorimotor contingencies, a world with different physical and interaction rules. An interesting example of this is work by the artist Char Davies who 15 years ago demonstrated a system where movement through the virtual world was via breath (Davies and Harrison, 1996). However, breaking away from natural sensorimotor contingencies could have implications for the time taken to adapt to this new world, from short term to long term adaptation and remapping. Even when requiring longer adaptation, it could eventually provide as vivid experience as in the real world. However, the implications for the time to re-adapt to the real world when leaving virtual reality, should also be considered. This is reminiscent of the famous experiments by George M. Stratton where a subject wore inverting lenses for several days and eventually adapted so that the vision seemed normal. However, after finally removing the lenses vision again inverted (Stratton, 1896).

## Virtual Objects

In order for perception and action in a virtual environment to be possible there have to be objects. Virtual objects can be considered at different levels. At one level, from the point of view of a programmer constructing a virtual environment, an object is a description in a data base consisting of geometry and material properties. The geometry specifies the shape of the object and the material how it emits or reflects light. There are likely to be other properties specifying physics, animation, collision responses and so on. During the actual execution of the program that leads to the display of the virtual environment, this database of objects is traversed by the computer typically at a minimum of 60 times a second and, given the set of objects and the current position and direction of gaze of the participant, the representations of the collection of objects is rendered onto the displays (at least two displays, one from a left and the other from a right eye point of view for stereoscopic vision). These displays are continuously updated as the viewpoint moves and gaze direction changes, causing the 2D projection images of objects to change according to perspective, and as a result of changes in visibility occlusions. It is much more complex than this of course because the objects may be animated. There are algorithms at work that determine light reflection between the objects, and light that reaches the positions in the environment corresponding to the eyes. This also computes visibility, i.e., which part of the scene is visible to the eye, and which occluded. If we go to a level below the specification and traversal of the objects comprising the environment, we could consider the particular electrical impulses that represent this process, or the electromagnetic profile represented in computer memory that corresponds to that object. If we want to go to a deeper level then we could consider, for example, the quantum aspects of the whole system.

If we step up to the level of the participant, then the objects may become *perceptual objects*. Through natural sensorimotor contingencies, head moves will allow a participant to, for example, see an object from different points of view and gaze directions, perceive it stereoscopically, move around it, bend underneath it, reach out to touch it, hear what happens as a result of a collision with it, even possibly see a reflection of the self (see below) in it. In an ideal-type immersive system, the object will have become an object of perception, and can be perceived following the rules of objects in reality: perception through bodily action. Since the whole environment consists of objects, the environment itself will become a “place.” In an ideal immersive system, sensory data, even though ultimately computer generated, will give rise to normal perceptual processes. The participant will have the illusion that there are objects there, in the same space as him- or herself, and the illusion of being in a place, other than the real-world place where all this is actually taking place (Sanchez-Vives and Slater, 2005). If perception is operating normally and coherent with body action (where natural sensorimotor contingencies are preserved) then the simplest hypothesis for the brain to form is that the virtual place is the real place where the participant is located, and that events there are actual events. This is the illusion of presence, which consists of the illusion of being in the place (Place Illusion) and the illusion of Plausibility (that events that appear to be happening are actually happening) (Slater, 2009). Of course, at a higher cognitive level, participants will know the true situation (for example, they will remember donning the equipment, and the equipment will have a weight on their body), but this cognitive knowledge interferes little with perception and with consequent responses. Confronted with a visual precipice immediately ahead in the VR, the safe thing to do is to step back from it, a deeply engrained self-protection mechanism associated to depth perception and already present in humans under 1 year of age (Gibson and Walk, 1960).

It follows from this that perceptions in VR are *first class*, meaning that they are perceptions at the same level as perceptions in reality (Chalmers, 2017). In practice they will have different properties—for example, objects may not be seen at the same high resolution as we see objects in the physical world with our real eyes. There may be some latency between head turns and updates to the display (if this is noticeable then it is likely that the whole illusion would break). The VR may not operate in all modalities—for example, without haptic feedback, so that occasionally there may be breaks in presence (Slater and Steed, 2000), corresponding to brief but significant moments when the illusion of presence is lost. The key is the extent to which sensorimotor contingencies operate naturally. This does not mean that events and situations portrayed in VR have to be realistic and follow the laws of physics. The issue is *how* they are perceived. Responses to the *content* that is perceived is in the domain of Plausibility, rather than Place Illusion. Plausibility is quite a complex issue, since when an environment is supposed to be a simulation of something that could really happen, then a tiny incorrect detail can destroy the illusion. For example, medical doctors in a virtual consultation were disturbed because they could not read from the virtual computer on their desk (Pan et al., 2016). However, people seem able to accept as really happening events that could never occur in reality—meaning that VR generates different expectations about situations and events. For example, in 3D chess played in VR pieces can fly across the board, and “this is how the laws of physics are in this world” (Slater et al., 1996). The scenario used for a fear of heights therapy described in Freeman et al. (2018) included a whale swimming in the air between buildings. Although participants found this strange, it did not break Plausibility.

It is worth considering for a moment the issue of color. From physics based computational models of light emission and reflection, the light reflected or emitted in every direction from every point on every virtual object can be computed. At any moment of time, only the finite set of rays of virtual light corresponding to those that reach the pixels on the display (and thus enter the eyes) needs to be computed. This computation then determines the light emitted from those pixels. This is what the participant *actually sees*—light emitting pixels of different wavelengths, typically modulated through a lens system. However, what the participant *perceives* are the colors of virtual objects, not the “objective reality” of the pixels. Looking at a virtual object the participant will see that object as a particular color and texture, see shadows projected onto it, and see its own shadow reflected on other surfaces. What the participant perceives is totally different from the objective reality of what the participant is actually seeing. The particular pattern of pixel colors gives rise to the perception of an illusory extended space and events within it, but it is this perception that is the basis of the conscious experience of the participant about where the participant is, and what is happening, what objects are there, what are their properties (e.g., shiny vs. matt), and the qualia associated with that experience.

Here we emphasize that what is *illusory* here is not the perception itself, which as we have indicated above is first class following (Chalmers, 2017). If a person sees a beautiful flower in VR they may have identical brain and behavioral responses to seeing a flower in reality. This perception itself is not illusory. However, the source of the perception is different in the two cases. In VR the perception arises from the interpretation of arrays of illuminated pixels as a flower located in extended space, a flower that the perceiver can walk around and admire from multiple viewpoints. The flower in reality is a physically existing object. From the moment that the light from the illuminated pixels in the case of the virtual flower reaches the eyes of the beholder, the perceptual process can be considered as the same as when the light from the real flower reaches the eyes. From this moment we can consider that the brain takes over, and can activate in the same way independently of the source of the light reaching the eyes. We refer to the perception of the virtual flower as illusory, not because the perception itself is illusory, but due to the source of the light. The perception itself is real, but the perceived flower is not real, from the standpoint of an observer outside of the VR.

## What Comes First, the Object or Consciousness of the Object?

While in neuroscience the debate is about the neural basis of consciousness and on whether consciousness only requires perception or perception and action—sensorimotor contingencies and goal-directed behavior (for a review see Storm et al., 2017; Pennartz, 2018), there are other theories that are more controversial. A theory crossing the bounds between philosophy and science is the Interface Theory of Perception (ITP) (Hoffman and Prakash, 2014). In this approach, perception is considered as an adaptive interface. Hoffman and Prakash (2014) argue that in reality we see only the payoffs of objects, not the objects as such (payoffs in an evolutionary sense). A claim of the ITP therefore is that our perceptions of the world are not veridical. Hoffman and Singh (2012) consider that the standard scientific view is that there is an “observer independent world” (OIW) and our perceptions of this are veridical representations. Using evolutionary game theory (Prakash et al., 2020) examined “the claim that the structure of conscious experience is, at least some of the time, homomorphic to the structure of the presumed OIW, and hence can be regarded as, at least some of the time, veridical in the strong sense required by a correspondence theory of truth.” Their results, however, show that survival in an evolutionary game depends not on veridical representation of reality, but that perceptions code for fitness payoffs. The ITP uses an analogy concerning the way we perceive “objects” on a computer screen such as icons, the waste bin, and so on. We know that the “reality” behind these is absolutely nothing like that which is perceived in the interface itself (the icon has a shape and color and size, but the underlying reality of the icon is totally different to this). However, we can carry out operations with the icon (such as “open a file”) that would be impossible to do operating directly on any level of the underlying reality.

So, when faced with, for example, a precipice in virtual reality, what we are seeing is not the “truth,” which is just a set of illuminated pixels that operate together with the brain’s visual system to result in the perception of a precipice, but the danger of falling that it implies. The “truth” corresponds at the end of the day (depending on how deep we want to go) to electromagnetic impulses at the root of the computer processing, and therefore in an entirely different domain to the virtual world.

VR, therefore, provides an even better exemplar of the ITP than the desktop interface—since VR can directly simulate “reality” itself. In VR when you are not perceiving an object, it does not exist in any ordinary meaning of the word “object.” This aspect, which in the real world has been a matter of philosophical discussion, in VR is literal, since the object does not exist even as a perceptual object unless the participant is actually looking at it. Staying with the visual domain, if the participant is not looking at an object, or more precisely if it does not fall within the participant’s visual field of view, it is not rendered onto the displays. It does not exist in the virtual world. However, it still does exist in the database (and all the levels below that that make the database physically possible), but these are not part of the virtual world. What we perceive in VR bears no resemblance to the “truth.”

## Space and Time in Virtual Reality

When we see a painting, a photograph, or while watching a video, we will have the impression of the objects depicted therein being arranged in a space. Of course, we know for sure that there is no actual space, and that everything is limited to the surface of the canvas, paper or screen. However, a VR experience is qualitatively different to the perception of a space depicted on a canvas or screen. Participants in VR do not simply “see” a space but they are “in” a space. It is indeed the most salient feature of VR, as we have discussed above, the idea of “Place Illusion.” What does it mean to be “in” a space? We can move through it. We can pick up objects and move them to a different location. We can walk around a cityscape or interact with other virtual characters. We can fly (Rosenberg et al., 2013). VR is increasingly referred to as a technology in the realm of “spatial computing.” VR computes and renders a space. Participants operate in that space much as they would in physical space. Yet, from our material perspective in physical reality we know absolutely that there is no space there. There are some light-emitting pixels on displays close to the eyes of the participant, and as the participant’s head moves around so the arrangement of wavelengths of light emitted from those pixels changes. Yet the participant has perceptual illusion of operating in an extended space, and corresponding qualia, the “feeling” to be in a space, and to be aware of having that feeling, and the qualia associated with seeing what is in the space—the objects, the colors, and so on. This movement through virtual spaces is even encoded in the “place cells” in the hippocampus, not only in humans (Ekstrom et al., 2003) but also in mice (Harvey et al., 2009) demonstrating that the “place illusion” is being encoded in the brain as physical navigation in real space (Brotons-Mas et al., 2006).

The perception of space arises wholly out of the brain’s interpretation of the patterns of light projected into the eyes as the gaze direction changes and optic flow is generated with movement. In physical reality we live in space and always experience spatial extension while conscious. Even most dreams are adventures through a space. The understanding of what space “really is” is of course a fundamental quest in physics, and our theories of space have changed over the generations. But in VR there is nothing to argue about—there is no space, only the light-emitting pixels—virtual space is completely subjective. Moreover, the physical basis of virtual space (the arrangement of light emitting pixels) is itself a product of human consciousness. The hardware and software were built by people, the particular patterns by which the pixels emit light as the gaze direction changes is totally governed by a computer program, itself the product of human consciousness. So “space” in VR is doubly the product of consciousness—its physical basis and construction is the product of consciousness, and the subjective awareness that it engenders is also the result of consciousness. Virtual space is therefore consciousness perceiving its own creation as if it were an objective reality.

In principle the same is true of time, although this has been studied much less in VR. By altering velocities of objects and self-location VR can be used to induce distortions in time perception, for example (Volante et al., 2018; Verde et al., 2019). VR can also be used to influence time in another way by inducing the illusion of traveling back through time (Friedman et al., 2014; Pizarro et al., 2015). This is based on an algorithmic solution to the Grandfather Paradox (Friedman, 2016). Having “lived” through a virtual experience, participants go back in virtual time, relive it once again, and see the actions of their previous self, doing whatever they did the first time around. By intervening, participants can change the future and experience an alternate history. Although not as obvious as space, changes in the perception of time can also be produced, yet examining this from the standpoint of material reality, of course there has been no alteration of time.

## Self-Perception

The perception of the self is more than just the consciousness of contents, as is the representation of the outside world, but is one of the building blocks of self-consciousness. What happens when a participant in a VR looks down toward his or her own real body? In the ideal-type immersive system they will see a virtual body that visually substitutes and is coincident in space with their own body. They will see this body from the normal first-person perspective that they see their own body in reality. With body tracking, they will notice that when they move their real body, the virtual body will move synchronously and correspondingly, limb by limb, providing correct visual, motor, and proprioceptive congruences. When a virtual object collides with their virtual body, it can be so arranged that they will feel the haptic information synchronously and correspondingly on their real body generating appropriate visuotactile correlations. When they look into a reflective surface, they will see a reflection of their virtual body. Their body will cast shadows, depending on the virtual light sources in the environment. This describes the physical set up that we refer to as “embodiment” (Spanlang et al., 2014).

Embodiment sets up a very strong prior for body self-perception. In our whole lives whenever we have looked down toward our body, we have seen our body, our interface to perceive and act in the world. Whenever we move we see our body move correspondingly. Whenever something touches us we feel it. When we look in a reflective surface we see our body. The shadow that follows us around is a shadow of our body. Therefore, the physics of embodiment gives rise to a third perceptual illusion beyond Place Illusion and Plausibility, which is the illusion of “body ownership,” the illusion that the virtual body is the actual body (Petkova and Ehrsson, 2008; Slater et al., 2010b). This is another perceptual illusion, since at the cognitive level, the participant knows for sure that it is not his or her real body, but it just feels as though it is. It is another qualia, hard to describe, only something that can be experienced.

This type of “body ownership illusion” is long known without exploiting VR, a paradigmatic example being the rubber hand illusion (RHI) (Botvinick and Cohen, 1998), though body ownership illusions were known prior to that (e.g., Lackner, 1988). In the RHI, the subject sits by a table with a rubber arm and hand in an anatomically plausible position in front, and the corresponding real arm and hand obscured behind a screen, but typically parallel to the rubber hand. The experimenter applies tactile stimulation to the real, unseen, hand (touching and stroking), and applies corresponding synchronous stimulation to the (seen) rubber hand, which is perceived visually in the same location where the real hand is being touched. From the point of view of the subject, a hand placed in a position so that it *could* be his or her hand is seen to be touched, while the corresponding actual touch is felt on the real hand. These two separate percepts are fused into one leading to the inference that the rubber hand is the subject’s hand. Proprioception shifts to the rubber hand, so for example, if the rubber hand is seen to be attacked the subject will react automatically and strongly to pull their corresponding real hand away, and there will be spike in physiological responses such as skin conductance. This same illusion has been shown to be engendered equally with a virtual hand in VR (Slater et al., 2008). With respect to the whole body a paradigm similar to the RHI was applied to a manikin body seen through streaming video from a pair of cameras mounted on top of the manikin to a HMD worn by the subject, so that the manikin body appeared to substitute the real body (Petkova and Ehrsson, 2008). The manikin and real body were tapped and stroked as in the RHI. Full model-based VR was used to show that the body ownership illusion also occurs for a whole virtual body, with visuotactile stimulation as in the RHI (Slater et al., 2010b), or visuomotor through real-time body tracking (Banakou and Slater, 2014).

Blanke et al. (2015) put forward a model where bodily self-consciousness relies on multisensory integration with corresponding proprioception and body-related information in other modalities, in particular visual. It has also been shown that auditory signals can be used to contribute to body representation illusions (Azañón et al., 2016; Tajadura-Jiménez et al., 2017). The multisensory integration must be within peripersonal space, so that it clearly and directly relates to the body. Blanke et al. (2015) argue that such multisensory integration results in body ownership, self-identification and the sense of location corresponding to the location of the virtual body. In the work described in this paper we always assume that the virtual body is virtually spatially coincident with the real body, but out-of-body illusions have been described where the virtual body may be in a different (virtual) location to where the viewpoint (and hence the real body) is located in the virtual space (Ehrsson, 2007; Lenggenhager et al., 2007). These requirements are fully met through the description of embodiment above. Moreover, following the Blanke et al. (2015) model, vestibular signals would be correct, for example, if the participant leans forward normal vestibular signals would be activated, since the real body is leaning forward, the visual flow would correspond to that act, and the virtual body would move accordingly with the lean in real-time. In fact, where vestibular signals break down is when the viewpoint of the participant is moved without corresponding body movements. In this case the most likely outcome is nausea.

The model proposed in Blanke et al. (2015) does not mention the appearance of the virtual body. To what extent does it have to look like the body of the actual participant? The answer seems to be that there is little constraint on the visual appearance of the virtual body for body self-consciousness. Evidence from the RHI suggests that the body needs to look humanoid, but adults can have strong body ownership over a child body (Banakou et al., 2013), an otherwise small body, a giant body (van der Hoort et al., 2011), a body of a different sex (Slater et al., 2010b) or race including a purple body (Peck et al., 2013; Banakou et al., 2016), a transparent body (Martini et al., 2015), an older body (Hershfield et al., 2011), and so on. The body may have additional limbs or a tail (Steptoe et al., 2013; Won et al., 2015), be asymmetric with one arm longer than the other (Kilteni et al., 2012), it may have an arm amputation (Kilteni et al., 2016), and so on. The precise constraints remain to be investigated but they appear to be very wide. Multisensory integration with respect to the virtual and real body dominates everything. In fact, comparing the level of subjective body ownership between a body that actually does look like the participant, and a different body that is much older has shown no difference in the level of subjective ownership in two different studies (Osimo et al., 2015; Slater et al., 2019).

A body ownership illusion has physiological, behavioral, attitudinal and cognitive consequences when the virtual body has physical or behavioral attributes that suggest such a change. Participants who see their virtual arm attacked will respond with brain activations that would be expected were their real arm attacked (Kilteni et al., 2012; González-Franco et al., 2013). Participants will have different pain thresholds to pain inflicted on their real body depending on their body representation in the VR (Martini et al., 2015; Matamala-Gomez et al., 2019a,b). Adults when embodied in a small virtual body will see the world as larger (van der Hoort et al., 2011), when embodied in what is specifically a child body will see the world on the average as around double the size of adults with an adult body that is the same size as the child (Banakou et al., 2013). It is the suggestive shape of the body being child-like that has this effect, not just the size. Embodying white people in a dark-skinned virtual body leads to a reduction in their implicit racial bias against black people (Peck et al., 2013; Maister et al., 2015; Banakou et al., 2016; Bedder et al., 2019), and participants in a dark-skinned virtual body are more likely to mimic the gestures of a black virtual partner than a white one (Hasler et al., 2017). The mimicry is important since it signifies rapport. At the behavioral level participants in a dark-skinned body informally dressed will play the drums with much greater body movement, than in a light-skinned and formally dressed body (in a between groups experiment) (Kilteni et al., 2013). At the cognitive level, participants embodied as Albert Einstein tend to have greater scores on a cognitive test, post- compared to a pre-test, than another group embodied in a body of the same approximate age as themselves (Banakou et al., 2018). Male domestic violence offenders will improve their fear recognition in the faces of women after being exposed to a situation of being embodied as a female subject to abuse from a male (Seinfeld et al., 2018). Men will be less aggressive to a virtual woman, compared to control groups, 1 week after experiencing sexual harassment from a group of men (Neyret et al., 2020). People embodied as Sigmund Freud tend to give better counseling advice to a body that looks like themselves than when they are the counselor in a body that is a copy of their own, or when embodied in the Freud body but without visuomotor synchrony (thus leading to much lower body ownership) (Osimo et al., 2015; Slater et al., 2019). Hence the type of body and the experience from the perspective of that body can influence how participants perceive and respond to events and situation in the (virtual world), and even their cognitive processing. A change in body representation in VR results in a change in bodily self-consciousness including conscious perception and cognition involving changes to aspects of the self.

## Is Consciousness First in Virtual Reality?

In VR participants perceive and act in a virtual world. But what is this world? As we have described above, the world in one form exists independently of the participant as a description in a database, that is then rendered by a computer program, where the rendering is determined by the visual field of view of the participant at any moment. There are similar constraints for auditory and haptic rendering. This gives rise to perceptual objects forming an environment, and it is this which is the world in which the participant operates. This virtual world does not exist independently of the perception of the participant, it is the perception and action of the participant that makes this world. To some extent, virtual reality is a realization of what the philosopher George Berkeley wrote in 1710: “The objects of sense exist only when they are perceived,” which echoes the popular question: “If a tree falls in a forest and no one is around to hear it, does it make a sound?” Even though these philosophical propositions are arguable, they are exactly the case in VR.

The ITP (Hoffman and Prakash, 2014) offers a formalism useful as a model for VR. In that theory, a “conscious agent” is defined by three entities: a World (W), conscious experiences (X) and actions (G). Mathematically, these are sets and their classes of subsets form events. There are probability measures (here simplified) *P*:*W*→*X* (perception), *D*:*X*→*G* (decision), and *A*:*G*→*W* (action). Hence an event in the World leads (probabilistically) to a perception, a perception leads to a decision, and a decision leads to an action that can have an effect in the World, which leads to perception, and so on.

In order to avoid the problem apparently inherent in this definition (i.e., that the world is independent of the conscious agent) the fundamental axiom of this theory is that “The world W consists entirely of conscious agents.” This is simply illustrated by a setup with two conscious agents. In this case the World of agent 1 consists of the actions of agent 2. Following the schema above, agent 1 makes a decision which then leads to its actions, which in turn form the World of agent 2. Agent 2 makes a decision leading to an action of agent 2, which is the world of agent 1, and so on. This can be extended to multiple agents, where, for example, the World of each agent is formed by the actions of all the other agents. The ITP then moves on to begin the process of proving mathematically that from this framework arises our normal world of object perception and all the laws of physics. For a recent extensive account, see Hoffman (2019). In other words, in this setup consciousness is primary, the world consists only and exclusively of conscious agents, and the world in every sense arises from interactions between them. There is no observer independent physical reality. Objects exist to the extent that they are perceived. The world of any conscious actor consists of the actions of all other conscious actors.

How does this apply to VR? We consider first the basic case where there is only one human agent in a virtual world. The virtual world itself as experienced by the participant is a product of human activity, i.e., the virtual world is “produced” by human consciousness. For example, if in the virtual environment the participant carries out an action of reaching toward an object to touch it, and thereupon the object changes color, every aspect of this has been programmed by someone (or by a team). This includes the representation of the body of the participant (there must at least be a representation of a hand otherwise nothing can be “touched”), the tracking of the hand and the rest of the body, the object itself—every aspect from its location in space to its appearance, the surrounding environment, the changing of the color and so on. Behind that, there is huge host of other human activity that created the underlying hardware and the software to interface to it, the manufacturing process, the delivery and assembly of all the items, the delivery of the hardware to the participants, even the advertising of the fact that the hardware exists and can be bought, the bank transfers involved, the infrastructure that makes the purchases possible, and so on. In terms of the human activity in the creation of the virtual environment in which the participant interacts one can envisage wave after wave of this spreading in ripples through a huge chunk of human activity. So, when a participant alone in a virtual environment perceives, decides and acts, at the very moment that this is done there may not be any other conscious agents with whom this interaction is taking place, but considered as a whole this event would have been impossible without the involvement of massive human agency. The program represents the foreseen interactions of the participant and responds as programmed (even if this includes responses based on pseudo-random choices). The “other agent” is there, only not synchronously except through the operation of a computer program.

If we now extend to multiple participants acting together in the same virtual environment, seeing and interacting with virtual representations of one another, then the formalism offered by the Interface Theory of Perception is an appropriate one. There is always one agent that is special, of course, which is the human agency that created the virtual environment. We call this Agent 0. We can conclude that indeed in the context of virtual environment, and keeping strictly to this level, and not considering the “true” situation (the computers and their electromagnetic impulses), that in VR consciousness is prime. Everything that happens and can happen is a product of human consciousness.

When multiple agents are introduced the question of object permanency becomes interesting. As argued by George Berkeley and also by ITP, an object does not exist when it is not perceived. Whatever we may think about this in the context of physical reality, in VR this is a true statement for perceptual objects—for example, unless something is in the visual field of view of the participant it will not be rendered. However, when there are multiple agents in the same virtual space, some may be looking toward the object and some not. So, for some participants the perceptual object exists and for others it does not. Presumably we have to say that an object exists in the virtual world if at least one agent is perceiving it.

Hoffman and Prakash (2014) prove mathematically that in their framework any subset of agents considered as an entity, also follows the formal definition of being a “conscious agent.” Here we briefly wish to consider a particular case of this, where there are two agents but they each represent the same person. In Osimo et al. (2015) and Slater et al. (2019) a participant plays two sides of a dialogue. They are embodied in a virtual body that is a scanned representation of themselves and also in a virtual body that represents Dr. Sigmund Freud. They sequentially swap between these two bodies, maintaining a dialogue between the two. First as the representation of themselves they explain a problem to Freud, and next when embodied as Freud they see and hear their self-representation explain the problem. They are then re-embodied in their self-representation and see and hear the reply of “Freud,” and so the dialogue continues with as many body swaps as the participant desires. In this situation, the participant is a conscious agent as him- or herself, is a conscious agent as Freud, from each perspective the actions of one form the world of the other. However, the two agents together also form a conscious agent, with the world created from the programmed actions of Agent 0. This provides an interesting perspective of what happens in this setup. Participants tend to find solutions to their personal problems when body swapping with the virtual Freud in this way, compared to control conditions. There is a split consciousness: from the point of view of the participants themselves, and from the point of view of the Freud virtual body. The affordance of this setup for personal problem solving has been explained through a combination of embodied perspective taking, as a manifest realization of “Solomons’ Paradox” where people generally make wiser decisions about themselves when thinking about these from the perspective of someone else rather than their own perspective (Grossmann and Kross, 2014). However, more than the interaction from the separate points of view of self and Freud, from the perspective of the ITP there is another level of consciousness, which is of the two considered as one entity. This is just another way of saying “the whole is more than the sum of the parts,” but it would be very interesting to transform this into a measurable hypothesis.

## Hypotheses

Here we consider some consequences of the above discussion in terms of experimental studies that have or could be carried out in VR in order to throw further light on whether consciousness is first in virtual environments.

In Banakou et al. (2013) adult participants were embodied in one of two virtual bodies: a child of about 5 years old, or an adult shaped body of the same height as the child. The embodiment was achieved through first person perspective over the virtual body and synchrony between their real body movements and the virtual body movements. They saw their virtual body both directly by looking down toward it and in a virtual mirror. This led to a high degree of subjective body ownership over both the child and the adult shaped bodies. In another condition there was asynchrony between real and virtual body movements leading to low subjective body ownership. Prior to the embodiment, participants had learned how to indicate the sizes of different cubes in the environment by the distance between their hands. After experiencing the embodiment, a number of cubes were measured again using the same method. It was found that for those in the synchronous embodied condition, on the average participants overestimated the sizes of the cubes. This finding was in line with a previous study that found embodiment of adults in a small doll led to overestimation of object sizes (van der Hoort et al., 2011). However, for those with the child body the overestimation of cube sizes was almost double that of those in the adult body. For the asynchronous conditions there were no effects. Perception of the cubes changed as a result of the shape of the body, not only its size. In this case a change in perception of the self as an object led to changes in perception of other objects. If the self was experienced as smaller and childlike, then this led to consequent changes in perception of the environment. Under these conditions the consciousness of the self, determined the perception of the external virtual world. The results of this study were later replicated (Tajadura-Jiménez et al., 2017). However, this is a reinterpretation of existing data.

Consider the following situation. A person starts at one corner of a virtual room which displays a number of pointed upright sticks and small tables, both of the same height. Their first task is to navigate to another corner diagonally across the room. Once they have done that, a large number of little cuboids (representing books) appear scattered over the floor. The person has the task to pick up all the books until that there are none left on the floor. The obvious solution is to pick up the books and place them on the tables. However, when a book is placed on any table the table becomes somewhat transparent and watery and the book sinks through and ends up on the floor again. If a book is placed on or near the top of a pointed stick in a radius of 0.5 m, it stays there. Over time the person picks up the many books and places them by the sticks thus completing the task, which brings a reward. This same procedure might be repeated many times by participants in an experimental study.

In this setup the payoffs for the sticks and tables have been changed, the books can rest on the sticks but not on the tables which have a watery aspect. As mentioned above, according to the ITP, perception is not veridical, but rather encodes payoffs that are useful for the perceiver. Following this, the perception of sticks and tables should therefore also change. At the end of picking up all the books from the floor, they all vanish, and the person is back in one corner of the room. They now have the task to navigate across back to the other corner. The arrangement of tables and sticks has changed, but there are still the same number. The navigation path, in particular the proximities to the sticks and tables can be computed and compared with the first navigation at the start of the experience. The question is whether participants navigate around the sticks as if they were wider than they are, ignoring the tables. This behavior would be evidence that perception of the sticks and tables had changed.

This experiment illustrates how VR can be used to test the extent to which perception of VR objects is encoding their “physical” properties or the “payoffs,” the integration of their role in the environment.

## Conclusion

In this paper we have discussed aspects of how consciousness of the (virtual) world and self-consciousness are generated in VR, considering relevant aspects of perception in VR. In VR, abstract objects exist independently of the participant at one level, but when viewed from within the context of VR, such objects that exist in a database become perceptual objects with which the participant can interact. The set of perceptual objects becomes the environment in which the participant perceives and acts. Objects that are not in at least one of the sensory fields-of-view of the participant, do not exist in the virtual world (they are not rendered even though they exist at many different levels in the physical world—for example, as data stored in computer memory). What is perceived in VR may not be real, but perception in VR is first class, in the sense that the perception itself is real. However, what is perceived is not “true.” The set of illuminated pixels (in turn caused by the operations of the computer and display devices at the back of any VR interface) is transformed into a perceived extended space that contains objects including a body representation of the participant. The space can be viewed from any perspective, objects can be touched, pushed, heard, in principle they can be even smelt, and so on, in an ideal-type immersive system. The question about the reality of what is perceived only emerges because the system is immersive.

Immersive systems form a partial order, where the relational operator is “immersion,” whereby one system is more “immersive” than another if the first can be used to simulate experiences of the second, but the second not the first. Each type of immersive system determines the range of possible perceptual experiences and actions, thereby giving rise to different sets of potential qualia. An ideal-type immersive system, at the “top” of this order, gives rise to qualia that may be indistinguishable from qualia induced by real world experiences: for example, a red cube perceived in reality may give rise to identical feelings when a red cube is perceived in virtual reality in the same kind of settings as in reality. A virtual flower may invoke similar feelings as a real flower. The most interesting aspect of this is that VR can be used to create scenarios that are impossible in reality, and thereby give rise to a new class of qualia that cannot otherwise be experienced. When we watch a movie, we do not doubt that the visual and auditory perception are real, but generated by a movie projector or through television. The remainder of the theater or living room is a continuous reminder of the non-reality of the displayed events, nevertheless sometimes evoking intense emotions. However, as immersive systems reach toward the top of the partial order the confusion with reality starts to blur, triggering new questions about the fundamental nature of reality and the role of consciousness.

Whether reality exists independently from the observer has been a subject of philosophical discussion. The Interface Theory of Perception provides a formalism for consciousness being primary. It defines conscious agents and removes an independently objective world by specifying that the world for any agent consists of the actions of all the other agents. When we consider a VR with only one participant, this framework leads to the conclusion that there is an Agent 0, which consists of all the human activity that leads to the computer program and hardware in which the VR experience is possible. The actions of Agent 0 appear in the virtual world through programming rather than being synchronously decided as a conscious entity during the running of the program. In this sense in VR consciousness is first i.e., primary. At the level of the virtual reality, there is no “objective reality” other than the activity of all conscious agents. The conscious agents determine the perceived world by their actions. Under these conditions, restricted to this virtual domain, consciousness is first.

With VR we create an alternate world, and then within it react to events that occur as if they were actually really happening to us. People create a virtual world where participants stand in front of a precipice (Meehan et al., 2002), or have to speak in front of a negative audience (Pertaub et al., 2002), and they become anxious. The constructed events of VR become reified, a transformation from conscious creations to objective realities.

The description of what takes place when someone experiences a virtual reality depends on the conceptual viewpoint and the level of analysis. From the “outside,” from the point of view of “matter is primary,” we know that the underlying reality is that a person in VR is looking at illuminated arrays of pixels which the perceptual system interprets as an extended space in which events occur and in which the person can act. We can then move to another level of analysis and consider the production of these illuminated arrays of pixels—the computer systems involved, the programming, the manufacturing and so on. All of these are themselves the products of human activity. If we stop at that level, we can argue that a VR experience is entirely the product of human consciousness. However, we can still go deeper, into both the physics that makes all this possible, and the functioning of the human perceptual system that leads to the interpretation. We can stop there or go still further to the level of electrical and chemical interactions between neurons, and eventually to the quantum level in physics, and so on. The Interface Theory of Perception argues that even these levels are themselves the product of consciousness. In one sense this is true—since these are scientific explanations and the science is the product of human consciousness (quantum physics is a theory, even though supported by a lot of evidence). Whether “matter” or “consciousness” are primary, i.e., with respect to the true nature of reality, is a different issue, and we are not proposing an answer to this philosophical question (which one day will have a scientific solution).

As we argued in Sanchez-Vives and Slater (2005) VR provides a highly useful paradigm from which to discuss the problem of understanding human consciousness. Here we have attempted to show that it acts an example of the notion that consciousness is first. In the domain of VR everything that happens, everything that can be experienced is the product of human consciousness. Perhaps by analogy this can help us to understand what this might mean in the domain of physical reality, even if we do not subscribe to the view that consciousness comes first.

## Author Contributions

Both authors listed have made a substantial, direct, and intellectual contribution to the work, and approved it for publication.

## Conflict of Interest

The authors declare that the research was conducted in the absence of any commercial or financial relationships that could be construed as a potential conflict of interest.

## Publisher’s Note

All claims expressed in this article are solely those of the authors and do not necessarily represent those of their affiliated organizations, or those of the publisher, the editors and the reviewers. Any product that may be evaluated in this article, or claim that may be made by its manufacturer, is not guaranteed or endorsed by the publisher.

## References

[B1] Al-Sada M. Jiang K. Ranade S. Kalkattawi M. Nakajima T. (2020). HapticSnakes: multi-haptic feedback wearable robots for immersive virtual reality. *Virtual Real.* 24 191–209. 10.1007/s10055-019-00404-x

[B2] Azañón E. Tamè L. Maravita A. Linkenauger S. A. Ferrè E. R. Tajadura-Jiménez A. (2016). Multimodal contributions to body representation. *Multisens. Res.* 29 635–661. 10.1163/22134808-00002531

[B3] Banakou D. Slater M. (2014). Body ownership causes illusory self-attribution of speaking and influences subsequent real speaking. *Proc. Natl. Acad. Sci. U.S.A.* 111 17678–17683. 10.1073/pnas.1414936111 25422444 PMC4267370

[B4] Banakou D. Groten R. Slater M. (2013). Illusory ownership of a virtual child body causes overestimation of object sizes and implicit attitude changes. *Proc. Natl. Acad. Sci. U.S.A* 110 12846–12851. 10.1073/pnas.1306779110 23858436 PMC3732927

[B5] Banakou D. Kishore S. Slater M. (2018). Virtually being einstein results in an improvement in cognitive task performance and a decrease in age bias. *Front. Psychol.* 9:917. 10.3389/fpsyg.2018.00917 29942270 PMC6004376

[B6] Banakou D. Pd H. Slater M. (2016). Virtual embodiment of white people in a black virtual body leads to a sustained reduction in their implicit racial bias. *Front. Hum. Neurosci.* 10:601. 10.3389/fnhum.2016.00601 27965555 PMC5126081

[B7] Bedder R. L. Bush D. Banakou D. Peck T. Slater M. Burgess N. (2019). A mechanistic account of bodily resonance and implicit bias. *Cognition* 184 1–10. 10.1016/j.cognition.2018.11.010 30553934 PMC6346146

[B8] Bitbol M. (2008). Is consciousness primary? *NeuroQuantology* 6 53–72. 10.14704/nq.2008.6.1.157

[B9] Blanke O. Slater M. Serino A. (2015). Behavioral, neural, and computational principles of bodily self-consciousness. *Neuron* 88 145–166. 10.1016/j.neuron.2015.09.029 26447578

[B10] Botvinick M. Cohen J. (1998). Rubber hands ‘feel’ touch that eyes see. *Nature* 391 756–756. 10.1038/35784 9486643

[B11] Brotons-Mas J. R. O’mara S. Sanchez-Vives M. V. (2006). Neural processing of spatial information: what we know about place cells and what they can tell us about presence. *Presence Teleoperators Virtual Environ.* 15 485–499. 10.1162/pres.15.5.485

[B12] Chalmers D. J. (1995). Facing up to the problem of consciousness. *J. Conscious. Stud.* 2 200–219.

[B13] Chalmers D. J. (1996). *The Conscious Mind: In Search Of A Fundamental Theory.* Oxford: Oxford university press.

[B14] Chalmers D. J. (2017). The virtual and the real. *Disputatio* 9 309–352. 10.1515/disp-2017-0009

[B15] Changeux J.-P. (1997). *Neuronal Man: The Biology Of Mind.* Princeton, NJ: Princeton University Press.

[B16] Crick F. Clark J. (1994). The astonishing hypothesis. *J. Conscious. Stud.* 1 10–16.

[B17] Davies C. Harrison J. (1996). Osmose: towards broadening the aesthetics of virtual reality. *ACM Siggraph Comput. Graph.* 30 25–28. 10.1145/240806.240808

[B18] Edelman G. M. (2003). Naturalizing consciousness: a theoretical framework. *Proc. Natl. Acad. Sci. U.S.A.* 100 5520–5524. 10.1073/pnas.0931349100 12702758 PMC154377

[B19] Edelman G. M. (2004). *Wider Than The Sky: The Phenomenal Gift Of Consciousness.* London: Yale University Press. 10.1172/JCI23795

[B20] Ehrsson H. H. (2007). The experimental induction of out-of-body experiences. *Science* 317:1048. 10.1126/science.1142175 17717177

[B21] Ekstrom A. D. Kahana M. J. Caplan J. B. Fields T. A. Isham E. A. Newman E. L. (2003). Cellular networks underlying human spatial navigation. *Nature* 425 184–188. 10.1038/nature01964 12968182

[B22] Freeman D. Haselton P. Freeman J. Spanlang B. Kishore S. Albery E. (2018). Automated psychological therapy using immersive virtual reality for treatment of fear of heights: a single-blind, parallel-group, randomised controlled trial. *Lancet Psychiatry* 5 625–632. 10.1016/S2215-0366(18)30226-8 30007519 PMC6063994

[B23] Friedman D. (2016). A computer program for simulating time travel and a possible’solution’for the grandfather paradox. *arXiv* [Preprint] arXiv:1609.08470,

[B24] Friedman D. Pizarro R. Or-Berkers K. Neyret S. Pan X. Slater M. (2014). A method for generating an illusion of backwards time travel using immersive virtual reality—an exploratory study. *Front. Psychol.* 5:943. 10.3389/fpsyg.2014.00943 25228889 PMC4151165

[B25] Gibson E. J. Walk R. D. (1960). The visual cliff. *Sci. Am.* 202 64–72. 10.1038/scientificamerican0460-64 13827949

[B26] González-Franco M. Peck T. C. Rodríguez-Fornells A. Slater M. (2013). A threat to a virtual hand elicits motor cortex activation. *Exp. Brain Res.* 232 875–887. 10.1007/s00221-013-3800-1 24337257

[B27] Grossmann I. Kross E. (2014). Exploring Solomon’s Paradox: Self-distancing eliminates the self-other asymmetry in wise reasoning about close relationships in younger and older adults. *Psychol. Sci.* 25 1571–1580. 10.1177/0956797614535400 24916084

[B28] Harvey C. D. Collman F. Dombeck D. A. Tank D. W. (2009). Intracellular dynamics of hippocampal place cells during virtual navigation. *Nature* 461 941–946. 10.1038/nature08499 19829374 PMC2771429

[B29] Hasler B. Spanlang B. Slater M. (2017). Virtual race transformation reverses racial in-group bias. *PLoS One* 12:e0174965. 10.1371/journal.pone.0174965 28437469 PMC5403166

[B30] Hershfield H. E. Goldstein D. G. Sharpe W. F. Fox J. Yeykelis L. Carstensen L. L. (2011). Increasing saving behavior through age-progressed renderings of the future self. *J. Mark. Res.* 48 S23–S37. 10.1509/jmkr.48.SPL.S23 24634544 PMC3949005

[B31] Hoffman D. (2019). *The Case Against Reality: Why Evolution Hid The Truth From Our Eyes.* New York, NY: WW Norton & Company.

[B32] Hoffman D. D. Prakash C. (2014). Objects of consciousness. *Front. Psychol.* 5:577. 10.3389/fpsyg.2014.00577 24987382 PMC4060643

[B33] Hoffman D. D. Singh M. (2012). Computational evolutionary perception. *Perception* 41 1073–1091. 10.1068/p7275 23409373

[B34] Hoffman D. D. Singh M. Prakash C. (2015). The interface theory of perception. *Psychon. Bull. Rev.* 22 1480–1506. 10.3758/s13423-015-0890-8 26384988

[B35] Kilteni K. Bergstrom I. Slater M. (2013). Drumming in immersive virtual reality: the body shapes the way we play. *IEEE Trans. Vis Comput. Graph.* 19 597–605. 10.1109/TVCG.2013.29 23428444

[B36] Kilteni K. Grau-Sánchez J. Veciana De Las Heras M. Rodríguez-Fornells A. Slater M. (2016). Decreased corticospinal excitability after the illusion of missing part of the arm. *Front. Hum. Neurosci.* 10:145. 10.3389/fnhum.2016.00145 27148005 PMC4830822

[B37] Kilteni K. Normand J.-M. Sanchez Vives M. V. Slater M. (2012). Extending body space in immersive virtual reality: a very long arm illusion. *PLoS One* 7:e40867. 10.1371/journal.pone.0040867 22829891 PMC3400672

[B38] Koch C. Massimini M. Boly M. Tononi G. (2016). Neural correlates of consciousness: progress and problems. *Nat. Rev. Neurosci.* 17 307–321. 10.1038/nrn.2016.22 27094080

[B39] Lackner J. R. (1988). Some proprioceptive influences on the perceptual representation of body shape and orientation. *Brain* 111 281–297. 10.1093/brain/111.2.281 3378137

[B40] Lenggenhager B. Tadi T. Metzinger T. Blanke O. (2007). Video ergo sum: manipulating bodily self-consciousness. *Science* 317 1096–1099. 10.1126/science.1143439 17717189

[B41] Maister L. Slater M. Sanchez-Vives M. V. Tsakiris M. (2015). Changing bodies changes minds: owning another body affects social cognition. *Trends Cogn. Sci.* 19 6–12. 10.1016/j.tics.2014.11.001 25524273

[B42] Manousakis E. (2006). Founding quantum theory on the basis of consciousness. *Found. Phys.* 36 795–838. 10.1016/j.pbiomolbio.2018.07.013 30076851

[B43] Martini M. Kilteni K. Maselli A. Sanchez-Vives M. V. (2015). The body fades away: investigating the effects of transparency of an embodied virtual body on pain threshold and body ownership. *Sci. Rep.* 5:13948. 10.1038/srep13948 26415748 PMC4586459

[B44] Matamala-Gomez M. Donegan T. Bottiroli S. Sandrini G. Sanchez-Vives M. V. Tassorelli C. (2019a). Immersive virtual reality and virtual embodiment for pain relief. *Front. Hum. Neurosci.* 13:279. 10.3389/fnhum.2019.00279 31551731 PMC6736618

[B45] Matamala-Gomez M. Gonzalez A. M. D. Slater M. Sanchez-Vives M. V. (2019b). Decreasing pain ratings in chronic arm pain through changing a virtual body: different strategies for different pain types. *J. Pain* 20 685–697. 10.1016/j.jpain.2018.12.001 30562584

[B46] Meehan M. Insko B. Whitton M. C. Brooks F. P. (2002). Physiological measures of presence in stressful virtual environments. *ACM Trans. Graph.* 21, 645–652. 10.1145/566654.566630

[B47] Melloni L. Mudrik L. Pitts M. Koch C. (2021). Making the hard problem of consciousness easier. *Science* 372 911–912. 10.1126/science.abj3259 34045342

[B48] Neyret S. Oliva R. Beacco A. Navarro X. Valenzuela J. Slater M. (2020). An embodied perspective as a victim of sexual harassment in virtual reality reduces action conformity in a later milgram obedience scenario. *Sci. Rep.* 10:6207. 10.1038/s41598-020-62932-w 32277079 PMC7148366

[B49] O’Regan J. K. Noë A. (2001a). A sensorimotor account of vision and visual consciousness. *Behav. Brain Sci.* 24 939–1031. 10.1017/s0140525x01000115 12239892

[B50] O’Regan J. K. Noë A. (2001b). What it is like to see: a sensorimotor theory of perceptual experience. *Synthese* 129 79–103.

[B51] Osimo S. A. Pizarro R. Spanlang B. Slater M. (2015). Conversations between self and self as Sigmund Freud – a virtual body ownership paradigm for self counselling. *Sci. Rep.* 5:13899. 10.1038/srep13899 26354311 PMC4564809

[B52] Pan X. Slater M. Beacco A. Navarro X. Swapp D. Hale J. (2016). The responses of medical general practitioners to unreasonable patient demand for antibiotics – a study of medical ethics using immersive virtual reality. *PLoS One* 11:e0146837. 10.1371/journal.pone.0146837 26889676 PMC4758661

[B53] Peck T. C. Seinfeld S. Aglioti S. M. Slater M. (2013). Putting yourself in the skin of a black avatar reduces implicit racial bias. *Conscious. Cogn.* 22 779–787. 10.1016/j.concog.2013.04.016 23727712

[B54] Pennartz C. M. (2018). Consciousness, representation, action: the importance of being goal-directed. *Trends Cogn. Sci.* 22 137–153. 10.1016/j.tics.2017.10.006 29233478

[B55] Pertaub D.-P. Slater M. Barker C. (2002). An experiment on public speaking anxiety in response to three different types of virtual audience. *Presence* 11 68–78. 10.1162/105474602317343668

[B56] Petkova V. I. Ehrsson H. H. (2008). If I were you : perceptual illusion of body swapping. *PLoS One* 3:e3832. 10.1371/journal.pone.0003832 19050755 PMC2585011

[B57] Pizarro R. Berkers K.-O. Slater M. Friedman D. (2015). “How to time travel in highly immersive virtual reality,” in *Proceedings of the 25th International Conference on Artificial Reality and Telexistence and 20th Eurographics Symposium on Virtual Environments*, (Goslar: Eurographics Association), 117–124.

[B58] Prakash C. Fields C. Hoffman D. D. Prentner R. Singh M. (2020). Fact, fiction, and fitness. *Entropy* 22:514. 10.3390/e22050514 33286286 PMC7517005

[B59] Rees G. Kreiman G. Koch C. (2002). Neural correlates of consciousness in humans. *Nat. Rev. Neurosci.* 3 261–270. 10.1038/nrn783 11967556

[B60] Rosenberg R. S. Baughman S. L. Bailenson J. N. (2013). Virtual superheroes: using superpowers in virtual reality to encourage prosocial behavior. *PLoS One* 8:e55003. 10.1371/journal.pone.0055003 23383029 PMC3559425

[B61] Sanchez-Vives M. V. Slater M. (2005). From presence to consciousness through virtual reality. *Nat. Rev. Neurosci.* 6 332–339. 10.1038/nrn1651 15803164

[B62] Seinfeld S. Arroyo-Palacios J. Iruretagoyena G. Hortensius R. Zapata L. E. Borland D. (2018). Offenders become the victim in virtual reality: impact of changing perspective in domestic violence. *Sci. Rep.* 8:2692. 10.1038/s41598-018-19987-7 29426819 PMC5807352

[B63] Slater M. (2009). Place Illusion and Plausibility can lead to realistic behaviour in immersive virtual environments. *Philos. Trans. R Soc Lond.* 364 3549–3557. 10.1098/rstb.2009.0138 19884149 PMC2781884

[B64] Slater M. Steed A. (2000). A virtual presence counter. *Presence* 9 413–434. 10.1162/105474600566925

[B65] Slater M. Linakis V. Usoh M. Kooper R. (1996). “Immersion, presence, and performance in virtual environments: an experiment with tri-dimensional chess,” in *Proceedings of the ACM Virtual Reality Software And Technology (VRST)*, Hong Kong, 163–172.

[B66] Slater M. Neyret S. Johnston T. Iruretagoyena G. Crespo M. ÁD. L. C. Alabèrnia-Segura M. (2019). An experimental study of a virtual reality counselling paradigm using embodied self-dialogue. *Sci. Rep.* 9:10903. 10.1038/s41598-019-46877-3 31358846 PMC6662659

[B67] Slater M. Perez-Marcos D. Ehrsson H. H. Sanchez-Vives M. (2008). Towards a digital body: the virtual arm illusion. *Front. Hum. Neurosci.* 2:6. 10.3389/neuro.09.006.2008 18958207 PMC2572198

[B68] Slater M. Spanlang B. Corominas D. (2010a). Simulating virtual environments within virtual environments as the basis for a psychophysics of presence. *ACM Trans. Graph.* 29:92.

[B69] Slater M. Spanlang B. Sanchez-Vives M. V. Blanke O. (2010b). First person experience of body transfer in virtual reality. *PLoS One* 5:e10564. 10.1371/journal.pone.0010564 20485681 PMC2868878

[B70] Spanlang B. Normand J.-M. Borland D. Kilteni K. Giannopoulos E. Pomes A. (2014). How to build an embodiment lab: achieving body representation illusions in virtual reality. *Front. Robot. AI* 1:9. 10.3389/frobt.2014.00009

[B71] Steptoe W. Steed A. Slater M. (2013). Human tails: ownership and control of extended humanoid avatars. *IEEE Trans. Vis. Comput. Graph.* 19 583–590. 10.1109/TVCG.2013.32 23428442

[B72] Storm J. F. Boly M. Casali A. G. Massimini M. Olcese U. Pennartz C. M. (2017). Consciousness regained: disentangling mechanisms, brain systems, and behavioral responses. *J. Neurosci.* 37 10882–10893. 10.1523/JNEUROSCI.1838-17.2017 29118218 PMC5678021

[B73] Stratton G. M. (1896). Some preliminary experiments on vision without inversion of the retinal image. *Psychol. Rev.* 3:611.

[B74] Tajadura-Jiménez A. Banakou D. Bianchi-Berthouze N. Slater M. (2017). Embodiment in a child-like talking virtual body influences object size perception, self-identification, and subsequent real speaking. *Sci. Rep.* 7:9637. 10.1038/s41598-017-09497-3 PMC5575082 28851953

[B75] Tononi G. Edelman G. M. (1998). Consciousness and complexity. *Science* 282 1846–1851. 10.3390/e23030293 9836628

[B76] van der Hoort B. Guterstam A. Ehrsson H. H. (2011). Being barbie: the size of one’s own body determines the perceived size of the world. *PLoS One* 6:e20195. 10.1371/journal.pone.002019 PMC3102093 21633503

[B77] Verde L. L. Alais D. Burr D. C. Morrone M. C. Macdougall H. Verstraten F. A. (2019). Time dilation effect in an active observer and virtual environment requires apparent motion: No dilation for retinal-or world-motion alone. *J. Vis.* 19:4. 10.1167/19.3.4 30896730

[B78] Volante W. G. Cruit J. Tice J. Shugars W. Hancock P. A. (2018). “Time flies: investigating duration judgments in virtual reality,” in *Proceedings of the Human Factors and Ergonomics Society Annual Meeting*, (Los Angeles, CA: SAGE Publications), 1777–1781. 10.1177/1541931218621403

[B79] Won A. S. Bailenson J. Lee J. Lanier J. (2015). Homuncular flexibility in virtual reality. *J. Comput. Med. Commun.* 20 241–259. 10.1111/jcc4.12107

